# Investigating the Influence of GABA Neurons on Dopamine Neurons in the Ventral Tegmental Area Using Optogenetic Techniques

**DOI:** 10.3390/ijms23031114

**Published:** 2022-01-20

**Authors:** Yasumi Ohta, Takaaki E. Murakami, Mamiko Kawahara, Makito Haruta, Hironari Takehara, Hiroyuki Tashiro, Kiyotaka Sasagawa, Jun Ohta, Metin Akay, Yasemin M. Akay

**Affiliations:** 1 Nara Institute of Science and Technology, 8916-5, Ikoma 630-0101, Japan; ohtay@ms.naist.jp (Y.O.); murakami.takaaki.mm5@ms.naist.jp (T.E.M.); mamikawa@ms.naist.jp (M.K.); m-haruta@ms.naist.jp (M.H.); t-hironarit@ms.naist.jp (H.T.); tashhiro@ms.naist.jp (H.T.); sasagawa@ms.naist.jp (K.S.); ohta@ms.naist.jp (J.O.); 2 Department of Biomedical Engineering, University of Houston, Houston, TX 77204-5060, USA; makay58@gmail.com

**Keywords:** optogenetics, GABA neurons, dopamine neurons

## Abstract

Dopamine (DA) is the key regulator of reward behavior. The DA neurons in the ventral tegmental area (VTA) and their projection areas, which include the prefrontal cortex (PFC), nucleus accumbens (NAc), and amygdala, play a primary role in the process of reward-driven behavior induced by the drugs of addiction, including nicotine and alcohol. In our previous study, we developed a novel platform consisting of micro-LED array devices to stimulate a large area of the brain of rats and monkeys with photo-stimulation and a microdialysis probe to estimate the DA release in the PFC. Our results suggested that the platform was able to detect the increased level of dopamine in the PFC in response to the photo-stimulation of both the PFC and VTA. In this study, we used this platform to photo-stimulate the VTA neurons in both ChrimsonR-expressing (non-specific) wild and dopamine transporter (DAT)-Cre (dopamine specific) mice, and measured the dopamine release in the nucleus accumbens shell (NAcShell). We measured the DA release in the NAcShell in response to optogenetic stimulation of the VTA neurons and investigated the effect of GABAergic neurons on dopaminergic neurons by histochemical studies. Comparing the photo-stimulation frequency of 2 Hz with that of 20 Hz, the change in DA concentration at the NAcShell was greater at 20 Hz in both cases. When ChrimsonR was expressed specifically for DA, the release of DA at the NAcShell increased in response to photo-stimulation of the VTA. In contrast, when ChrimsonR was expressed non-specifically, the amount of DA released was almost unchanged upon photo-stimulation. However, for nonspecifically expressed ChrimsonR, intraperitoneal injection of bicuculline, a competitive antagonist at the GABA-binding site of the GABA_A_ receptor, also significantly increased the release of DA at the NAcShell in response to photo-stimulation of the VTA. The results of immunochemical staining confirm that GABAergic neurons in the VTA suppress DA activation, and also indicate that alterations in GABAergic neurons may have serious downstream effects on DA activity, NAcShell release, and neural adaptation of the VTA. This study also confirms that optogenetics technology is crucial to study the relationship between the mesolimbic dopaminergic and GABAergic neurons in a neural-specific manner.

## 1. Introduction

Dopamine (DA) is the key regulator of the reward behavior; DA neurons are present in the ventral tegmental area (VTA) with projection areas including the prefrontal cortex (PFC), nucleus accumbens shell (NAcShell), and amygdala. DA is thought to be very important in the process of reward-driven behavior induced by the drugs related to addiction, including nicotine and alcohol. Addictive substances (e.g., nicotine and alcohol) alters the reward system in the brain by modulating its activity and causing an increase of DA release from the VTA to the PFC and NAcShell. The mesocorticolimbic DA system, known as the reward circuit in the brain, plays a key role in this reward system as addictive substances trigger DA release through this system [1]. This pathway/system facilitates the reinforcing and/or withdrawal properties of addictive substances [1,2,3]. Previous studies suggested that nicotine exposure induces neurotransmitter function through the stimulation of DA neurons within the VTA, which mediate the release of DA and cause an increased neuronal firing along its projection pathways [4]. Systemic nicotine exposure enhances DA release within the NAcShell through the stimulation of VTA DA neurons [5,6,7,8,9]. The VTA mainly consists of DA, γ-aminobutyric acid (GABA), and glutamate neurons, with some neurons displaying combinatorial neurotransmitter characteristics. Within the VTA, nicotine directly binds and activates nicotinic acetylcholine receptors (nAChRs), which are ligand-gated ion channels found within the central and peripheral nervous system. These receptors are abundant in the VTA neurons. Therefore, nicotine directly activates DA neurons and indirectly activates glutamate and GABA neurons, enhancing DA release within the NAcShell [10,11,12]. Previous studies also suggest that GABAergic neurons regulate DA neurons [13,14,15]. A recent study shows that the inter-play between DAergic and GABAergic transmission presents novel relevant mechanisms in reinforcing addiction to alcohol and other addictive drugs [16].

In a previous study, we used GCaMP transgenic mice to observe DA neural activities to monitor neural activities within the VTA, PFC and NAcShell. GCaMP fluorescence and DA release reactions related to nicotine intake were measured simultaneously. Our data suggested that GCaMP fluorescence in the VTA and DA release in the PFC and NAcShell associated with nicotine intake can be measured simultaneously by a novel platform that combines an implantable microimaging device and a microdialysis probe [17].

In another recent study, we have developed a novel platform consisting of micro-LED array devices to stimulate a large-area of the brain of rats and monkeys with photo-stimulation (PS) and a microdialysis probe to estimate the DA release in the PFC and the NAcShell. Our results suggested that the platform was able to detect the increased level of DA in the PFC in response to the PS of both the PFC and VTA. PS devices, integrated with microdialysis probes for neurotransmitter release investigation in rats, were used to constitutively express ChrimsonR. In order to demonstrate the effectiveness of micro-LED array devices for PS over wide areas, first, we expressed ChR2 in the VTA of a macaque monkey. The DA released in the PFC was detected via microdialysis following the PS. A linear-type PS device with red LEDs was used in the rat experiments. By expressing ChrimsonR in the rat VTA and combining PS with microdialysis, we obtained promising results on the projection of dopaminergic neurons from the VTA to the PFC [18]. 

In this study, we investigated the influence of GABA neurons on DA neurons by measuring the DA release in the NAcShell following PS of the VTA in both ChrimsonR-expressing wild (non-specific) and dopamine transporter (DAT)-Cre (dopamine specific) mice which are genetically engineering mouse lines. For the study of DA neurons, promoter sequences driving expression of the *DAT* gene are widely used to direct the gene encoding Cre-recombinase activity to DA neurons [19,20]. In some of our experiments in this study, we focused only on DA neural activity by using DAT-Cre mice, which express Cre-recombinase under the control of the endogenous DA transporter promoter, thus enabling specific expression of Cre-recombinase in DA neurons. For wild (non-specific) mice, bicuculline was injected intraperitoneally to further investigate the release of DA at the NAcShell in response to PS of the VTA. Bicuculline was selected since it is a competitive antagonist at the GABA-binding site of the GABA_A_ receptor [21]. The influence of GABAergic neurons on DA activity and NAcShell release was confirmed using immunochemical staining.

## 2. Results

### 2.1. Investigation of Mesolimbic DA Neural Effects Using Optogenetics Techniques

**Figure 1:**
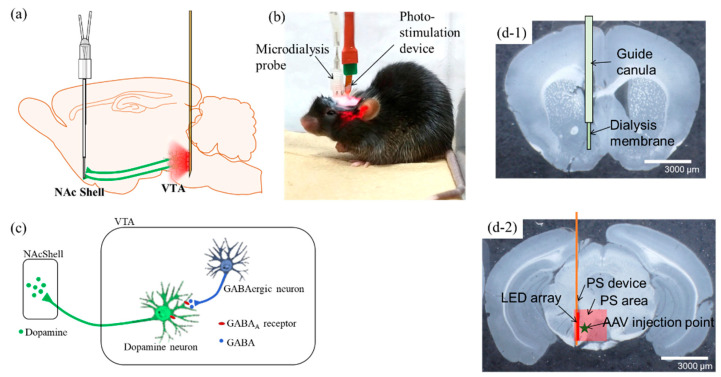
Conceptual diagram and implantation of the photo-stimulation (PS) device and the microdialysis probe in the mouse brain. (**a**) Conceptual diagram of the mesolimbic system, which extends axons from cell bodies in the VTA of the midbrain to the NAcShell. DA neurons expressing ChrimsonR are photo-stimulated at the wavelength of 620 nm, and the concentration of dopamine released in the NAcShell is measured by microdialysis. (**b**) Photograph of a mouse undergoing PS under freely moving conditions. A cable for PS and a tube for microdialysis are attached to the head of the mouse. Both are lightweight and do not interfere with the behavior of the mouse. (**c**) A simplified diagram showing the neural connections between dopaminergic and GABAergic neurons in the VTA and the release of DA in the NAcShell projecting from the cell body. The DA neurons in the VTA are inhibited by GABAergic neurons also in the VTA. Glutamatergic neurons from other sites acting on dopaminergic neurons are omitted. (**d-1**) Photomicrograph of a coronal slice of mouse brain in the NAcShell, showing the position of the microdialysis probe superimposed. (**d-2**) Photomicrograph of a coronal slice of mouse brain in the VTA, showing the implantation position of the PS device, the LED array area (red line in the figure), the LED irradiation area (light red square in the figure), and the AAV injection position (green star in the figure) superimposed. Scale bars: 3000 µm in (**d-1**,**d-2**). Abbreviations: NAc, nucleus accumbens; VTA, ventral tegmental area; DA: dopamine; AAV, adeno-associated virus; PS, photo-stimulation.

The mesolimbic system and its impact on the reward system in alcohol and nicotine addiction has been vigorously investigated. In this study, we aimed to elucidate this phenomenon using optogenetics techniques, by exploiting dopaminergic neurons in the VTA which are controlled by GABAergic neurons. Figure 1a shows a schematic diagram of DA neurons extending its axon from the VTA to the NAcShell. Neurons expressing ChrimsonR and located in the VTA of transgenic mice were activated with red light (620 nm) using a PS device implanted in the VTA. The same mice were implanted with a microdialysis probe in the NAcShell (Figure 1b), which is the projection site of VTA dopaminergic neurons. The amount of DA release induced by PS in the NAcShell was measured over time using high-performance liquid chromatography (HPLC). Mice were maintained under freely moving conditions. A simplified schematic of the relationship between DA and GABA neurons in the VTA is illustrated in Figure 1c.

The attached movie shown in the supplement demonstrates that our LED-based PS device and microdialysis probe are light-weight and small, and thus do not interfere with the behavior of the mice.

**Figure 2:**
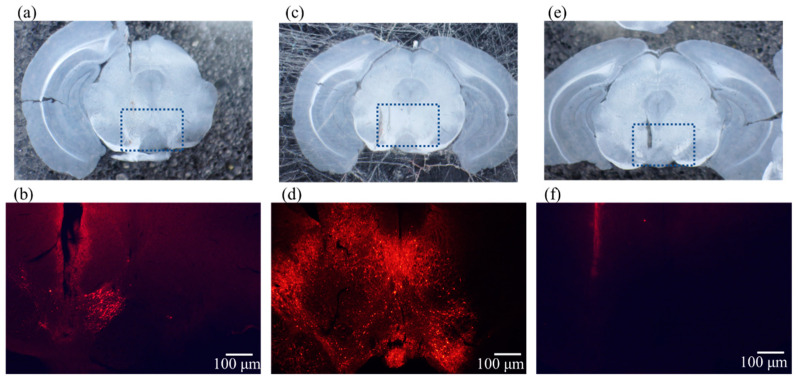
Coronal slice images including the VTA region around 3.2 mm posterior (upper panel). The dotted box marks the area of fluorescence micrographs of AAV-ChrimsonR expression (lower panel) from the corresponding image above; the scale bar is 100 µm. The red fluorescent neurons in the lower panel indicate neurons where ChrimsonR is expressed because mScarlet is expressed as a marker downstream of ChrimsonR. (**a**). Photograph of a section containing the VTA region of DAT-Cre mouse expressing ChrimsonR in the VTA. (**b**). Fluorescence micrographs show that only dopaminergic neurons in the VTA are specifically expressed. (**c**). Photographs depict the section containing the VTA region of a wild-type mouse expressing ChrimsonR in the VTA. (**d**). Fluorescence micrographs show that ChrimsonR is expressed not only in dopaminergic but also in non-dopaminergic neurons. (**e**). Photographs show the section containing the VTA region of a wild-type mouse as the control. (**f**). Fluorescence micrographs indicate that ChrimsonR is not expressed. Abbreviations: VTA, ventral tegmental area; AAV, adeno-associated virus.

After the experiment was completed, the brain was perfusion-fixed, removed and sectioned to confirm the position of the implant. Figure 1(d-1) shows a brain section of the NAcShell, together with a microdialysis probe collecting a sample from the tip of the guide cannula with 1 mm of dialysis membrane exposed. Figure 1(d-2) shows a brain section from the VTA with an implanted PS device. The positions of the AAV injection of ChrimsonR, PS device, LED array, and photo exposure area are superimposed on the section image.

### 2.2. Expression Results of AAV-ChrimsonR

ChrimsonR, a photosensitive channelrhodopsin protein, was introduced to the VTA by AAV injection. Since the aim of this study was to elucidate the regulation of DA neurons by GABAergic neurons in the VTA using optogenetic techniques, we prepared mice with three different expression patterns.

First, we injected EF1a-DIO-Chrimson-R-mRuby2-KV2.1 into the VTA of DAT-Cre mice to specifically activate DA neurons. Figure 2a shows an image of the coronal slice containing the VTA region, and Figure 2b shows a fluorescence microscopy image of the region marked with dotted lines in the corresponding photograph above. The red fluorescence marks the presence of mRuby2, which is expressed as a marker downstream of ChrimsonR, and is consistent with the localization of DA neurons in the injected hemisphere.

Next, to express ChrimsonR in all neurons in the VTA including GABAergic neurons, we injected CamKIIa-ChrimsonR-mScarlet-KV2.1 into the VTA of wild-type (WT) mice. Figure 2c shows an image of the coronal slice containing the VTA region, and Figure 2d shows a fluorescence microscopy image of the area enclosed by the dotted line in the above photograph. The red fluorescence indicates the expression of mScarlet, a marker downstream of ChrimsonR, in a large area of the VTA of the injected hemisphere.

Finally, for the control group, we used WT mice, which do not express ChrimsonR. Similarly, corresponding sections and fluorescence microscopy images are shown in Figure 2e,f, respectively, showing that ChrimsonR is not expressed in these WT mice.

### 2.3. Optogenetics-Microdialysis Experiment

**Figure 3:**
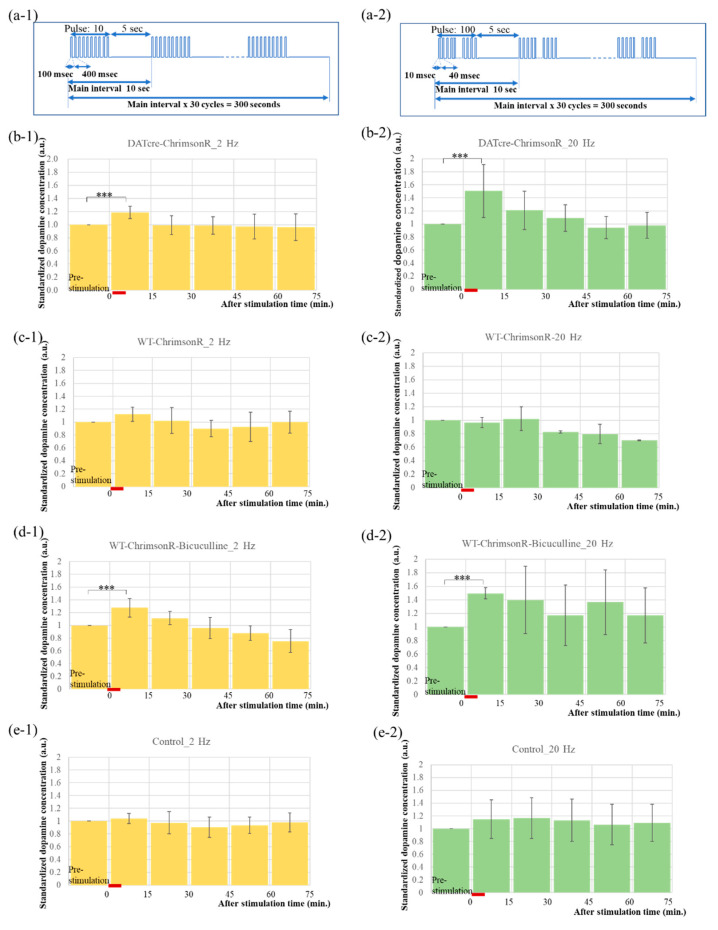
Photo-stimulation conditions by a PS device and the results of dopamine concentration measurement by microdialysis experiments. The red line each graph shows the time of the PS. (**a-1**,**a-2**) PS pulse conditions. The current is 3 mA, and the duty ratio is 20% in both cases. The frequencies are 2 Hz and 20 Hz in (**a-1**,**a-2**), respectively. (**b-1**,**b-2**) Time-lapse results of DA concentration at the NAcShell in the DAT-Cre mice which were injected with AAV-ChrimsonR into the VTA under the PS pulse conditions, as shown in (**a-1**,**a-2**). The bar graphs show that the DA concentration increase after PS at both 2 Hz and 20 Hz. The P-values of the DA concentration ratios of the dialysis samples for 15 min immediately after PS were 0.000116 and 0.00599, respectively. A red line indicates the PS period in 5 min in the graphs from (**b**) to (**e**). The height of the bars in the bar graph of (**b**) to (**e**) show the DA concentration ratio with respect to the pre-stimulus mean, respectively, and the error bars show its standard deviation. and the respective values are shown in Table 1. (**c-1**,**c-2**) Time-lapse results of DA concentration at the NAcShell in the wild-type mice injected AAV-ChrimsonR into the VTA shown in the same way as (**b**). The bar graphs show that the DA concentration does not increase after PS at 2 Hz and 20 Hz. The p-values of the DA concentration ratios of the dialysis samples for 15 min immediately after PS were 0.0677 and 0.416, respectively. (**d-1**,**d-2**) Time-lapse results of DA concentration at the NAcShell in the wild-type mice injected with AAV-ChrimsonR into the VTA shown in the same way as (**b**). These mice were given GABA receptor antagonist bicuculline to the abdomen 15 min before PS. The bar graphs show that the DA concentration increase after PS at both 2 Hz and 20 Hz. The P-values of the DA concentration ratios of the dialysis samples for 15 min immediately after PS were 0.00452 and 0.000243, respectively. (**e-1**,**e-2**) Time-lapse results of DA concentration at the NAcShell in the wild-type mice (i.e., control) without injection of ChrimsonR shown in the same way as (**b**). DA concentration does not increase after PS at 2 Hz and 20 Hz. The p-values of DA concentration ratios from the dialysis samples for 15 min immediately after PS were 0.0923 and 0.129, respectively. Abbreviations: NAc, nucleus accumbens; VTA, ventral tegmental area; DA: dopamine; AAV, adeno-associated virus; PS, photo-stimulation. *** indicates *p* < 0.001.

The pulse conditions of the PS are shown in Figure 3(a-1,a-2). In Figure 3(a-1), a pulse with frequency of 2 Hz and duty ratio d = 20% was maintained on for 5 s, then off for 5 s. This set (total time = 10 s) was performed 30 times for a total of 5 min. In Figure 3(a-2), the pulse frequency was increased to 20 Hz. Three LEDs were connected in series and driven at 3 mA. These conditions maintained the light intensity of 1 mW/mm^2^ required to open the channel of ChrimsonR without causing damage to brain tissue from heat [22,23].

Figure 3 shows the results of changes in DA concentration at NAcShell over time with a 2 Hz stimulus on the left side and a 20 Hz stimulus on the right side. Figure 3b shows the PS for a DAT-Cre mouse that expresses ChrimsonR specifically for DA neurons. EF1a-DIO-Chrimson-R-mRuby2-KV2.1 is injected into VTA of this DAT-Cre mouse. Figure 3c shows the PS for a WT mouse in which CamKIIa-Chrimson-R-mScarlet-KV2.1 was injected into VTA and stimulated non-specifically to DA neurons. Figure 3d shows the similar mice as in Figure 3c, but injected with bicuculline, an antagonist of GABA receptor, in the abdomen 15 min before the PS. Figure 3e is a control experiment in which WT mice, which do not express ChrimsonR at all, were subjected to the same PS. The red lines in Figure 3b–e show the timing of PS.

In the case of DA-specific PS, DA concentration increased immediately after PS, specifically shown in Figure 3b. In addition, the effect of PS was more pronounced at 20 Hz than at 2 Hz. In contrast, DA concentrations did not change even immediately after PS in a DA-nonspecific manner, as shown in Figure 3c. Furthermore, when GABA binding to GABA receptors in DA neurons in the VTA was blocked by bicuculline, DA concentration increased immediately after stimulation at both 2 Hz and 20 Hz, as shown in Figure 3d. Again, the effect appears to be more pronounced at 20 Hz than at 2 Hz. Finally, Figure 3 shows that the DA concentration remained almost unchanged regardless of PS in the control experiment, suggesting that it is not affected by LED irradiation, which is consistent with our previous data [18]. 

**Figure 4:**
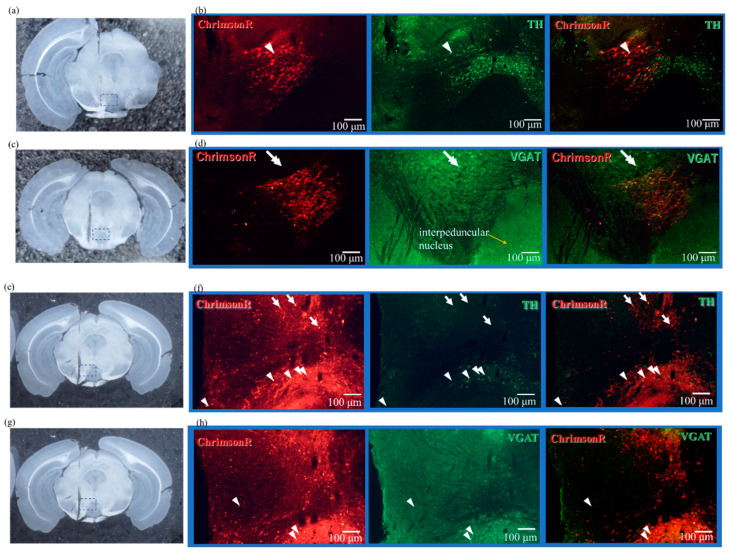
Fluorescence micrographs of ChrimsonR expression and immunostaining of DA and GABA neurons. The dotted box marks the area of fluorescence micrographs from the corresponding image on the left; the scale bar is 100 µm. (**a**,**c**) Photomicrographs of coronal slices around 3.2 mm posterior of mouse brain expressing ChrimsonR in DAT-Cre mice. (**b**) Fluorescence micrographs of the section shown in (**a**), showing a magnified image of the area indicated by the dotted line. The left end shows fluorescence micrographs of ChrimsonR expression, and the center shows fluorescence micrographs immunostained with anti-TH antibody showing dopaminergic cell bodies. The rightmost image is an overlay of the two images. As an example, the cell body shown with arrowhead is overlay of the cells stained with ChrimsonR and TH antibody, indicating that ChrimsonR is expressed in dopaminergic neurons. (**d**) Fluorescence micrograph of the section shown in (**c**), showing a magnified image of the area indicated by the dotted line. The left end shows the expression of ChrimsonR, and the center shows the cell body of GABAergic neuron immunostained with anti-VGAT antibody. The rightmost image is an overlay of them. As an example, the cell bodies indicated by double arrows are GABAergic cell bodies, but they do not overlap with ChrimsonR expression. On the other hand, there is an overlap in the region where ChrimsonR is expressed, but there is little overlap of clear GABAergic neurons, indicating that there are GABAergic cell bodies very close to dopamine neurons. (**e**,**g**) Photomicrographs of coronal slices around 3.2 mm posterior of WT-type mouse brain expressing ChrimsonR. (**f**) Fluorescence micrograph of the section shown in (**e**) showing a magnified image of the area indicated by the dotted line. The left end shows fluorescence micrographs of ChrimsonR expression, and the center shows fluorescence micrographs of the dopaminergic cell bodies immunostained with anti-TH antibody. The rightmost image is an overlay of the two images. As an example, the cell bodies shown with arrowheads are overlays of cells stained with ChrimsonR and TH antibody, indicating that ChrimsonR is expressed in dopaminergic neurons. On the other hand, the cell bodies, indicated by arrows, which are not stained with TH antibody, pointing out that ChrimsonR is also expressed in other areas than dopaminergic neurons. (**h**) Fluorescence micrograph of the section shown in (**g**), depicting a magnified image of the area indicated by the dotted line. The left end shows the expression of ChrimsonR, and the center shows the cell body of GABAergic neuron immunostained with anti-VGAT antibody. The rightmost image is an overlay of them. As an example, the cell bodies indicated by arrows are GABAergic cell bodies and overlap with the expression of ChrimsonR, indicating that ChrimsonR is also expressed in GABAergic neurons. All scale bars are 100 µm. Abbreviations: DA: dopamine.

Figure 4a,c show the coronal slices around the VTA of DAT-Cre mice injected with EF1a-DIO-ChrimsonR-mRuby2-KV2.1 to express DA-specific ChrimsonR. Figure 4b,d are fluorescence microscopy images of the area indicated by the dotted line in Figure 4a,c, respectively. The arrowhead in Figure 4b marks where ChrimsonR is expressed in the DA neuron. In contrast, the double arrows in Figure 4d show that ChrimsonR is not expressed in GABAergic neuron. In the right panel of Figure 4d, there is a yellow area where ChrimsonR and GABAergic neuron seem to overlap, but a closer examination reveals that there is little overlap of cell bodies, suggesting that the axons of GABAergic neuron overlap.

### 2.4. Results of Immunostaining Experiments for Expression of ChrimsonR in VTA

Whether ChrimsonR is expressed in DA neuron or GABAergic neuron in VTA is an important point in this paper. Coronal slices of the VTA of mice are shown in Figure 4a,c,e,g. These show the position of the PS device within the brain tissue.

Coronal slices from the VTA of WT mice injected with CamKIIa-ChrimsonR-mScarlet-KV2.1 and expressing DA-nonspecific ChrimsonR, presented in Figure 4e,g and Figure 4f,h, show immunostained fluorescence microscopy images of the dotted lines in Figure 4e,g, respectively. In Figure 4f,h, the left image shows the expression of ChrimsonR, the middle image is the fluorescence microscopy image immunostained with TH antibody and VGAT antibody, respectively, and the right image is the overlay of these images. In Figure 4f, arrowheads indicate where ChrimsonR overlaps with TH antibody. This indicates that ChrimsonR is expressed in the DA neuron. In Figure 4f, the arrow indicates the position where ChrimsonR does not overlap with TH antibody. This suggests ChrimsonR is expressed in DA and other neurons. In Figure 4h, the arrowhead shows the overlap between ChrimsonR and VGAT antibody, which indicates that ChrimsonR is expressed in GABAergic neuron. In particular, the lower part of Figure 4h is the interpeduncular nucleus, where many GABAergic neurons are distributed. Figure 4f,h show that ChrimsonR is expressed in both the DA neuron and GABAergic neuron.

### 2.5. Confirmation of Neural Activity with C-Fos Antibody

**Figure 5:**
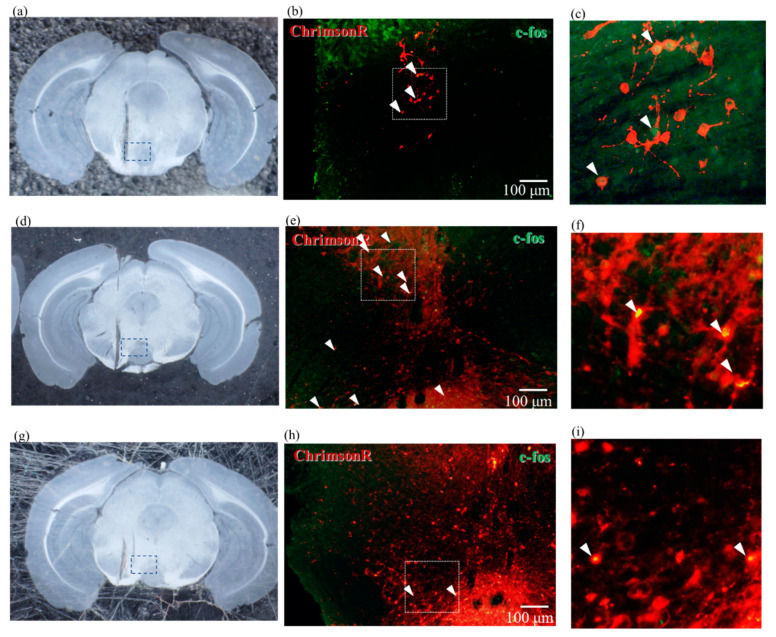
Fluorescence superimposed micrographs of ChrimsonR expression and immunostaining image against c-fos. The dotted box marks the area of fluorescence micrographs from the corresponding image on the left; the scale bar is 100 µm. (**a**) Photomicrograph of a coronal slice around 3.2 mm posterior of the mouse brain expressing ChrimsonR in DAT-Cre mice. (**b**) Fluorescence micrograph of the section shown in (**a**) with the dotted line magnified; neural cell bodies expressing ChrimsonR are stained red, and those immunostained with an anti c-fos antibody are shown in green. The overlap of the two images is clearly visible at the arrowhead. (**c**) Enlarged view of the area enclosed by the dotted line in (**b**). The center of the cell body, which is stained red and expresses ChrimsonR, and is stained green and expresses c-fos, indicating that the neuron is active. (**d**) Photomicrograph of a coronal slice around 3.2 mm posterior of a mouse brain expressing ChrimsonR in wild-type mice. (**e**) Magnified fluorescence micrograph of the section indicated by the dotted line in (**d**). The arrowhead shows many cell bodies immunostained with anti c-fos antibody overlap with the neuronal cell body where ChrimsonR is widely expressed. (**f**) Enlarged view of the area enclosed by the dotted line in (**e**). C-fos is expressed in a wide range of cell bodies where ChrimsonR is expressed, indicating that neurons are active. (**g**) Photomicrograph of a coronal slice around 3.2 mm posterior of a mouse brain expressing ChrimsonR in wild-type mice that had been peritoneally treated with bicuculline. (**h**) Fluorescence micrograph of the dotted area of the section shown in (**g**), showing that the neuronal cell bodies expressing ChrimsonR are stained red, and those immunostained with an anti c-fos antibody are shown in green. The overlap of the two images is marked by the arrowhead. (**i**) Enlarged view of the area enclosed by the dotted line in (**h**). The center of the cell body, which is stained red and expresses ChrimsonR, and is stained green and expresses c-fos, indicating that the neuron is active.

To confirm whether ChrimsonR is activated by PS, we performed immunostaining using c-fos antibody. Figure 5a is a photograph of a coronal slice near the VTA of DAT-Cre mice injected with EF1a-DIO-ChrimsonR-mRuby2-KV2.1 which expresses DA-specific ChrimsonR. Figure 5b is a fluorescence micrograph of the dotted area in Figure 5a immunostained with c-fos antibody. Figure 5c is a magnified image of Figure 5b, showing that the green fluorescently stained c-fos is overlaid and co-localized with red ChrimsonR by arrowheads. This result indicates that DA neuron is activated by PS. Figure 5d shows a coronal slice near the VTA of WT mice injected with CamKIIa-ChrimsonR-mScarlet-KV2.1 and expressing DA-nonspecific ChrimsonR. The arrowhead in Figure 5e shows the activity of ChrimsonR as in Figure 5b, and it is more widely expressed than in Figure 5b. Figure 5g shows the coronal slice near the VTA of a WT mouse injected with CamKIIa-Chrimson-R-mScarlet-KV2.1 and photo-stimulated in a DA-nonspecific manner. In this case, bicuculline, an antagonist of GABA receptor, was injected into the abdomen 15 min before PS. Although DA is non-specifically photo-stimulated, bicuculline prevents DA neurons in the VTA from interacting with GABAergic neurons. The arrowhead in Figure 5h shows the localization of the DA neuron as in Figure 5b,e and ChrimsonR on the DA neuron is active. 

**Figure 6:**
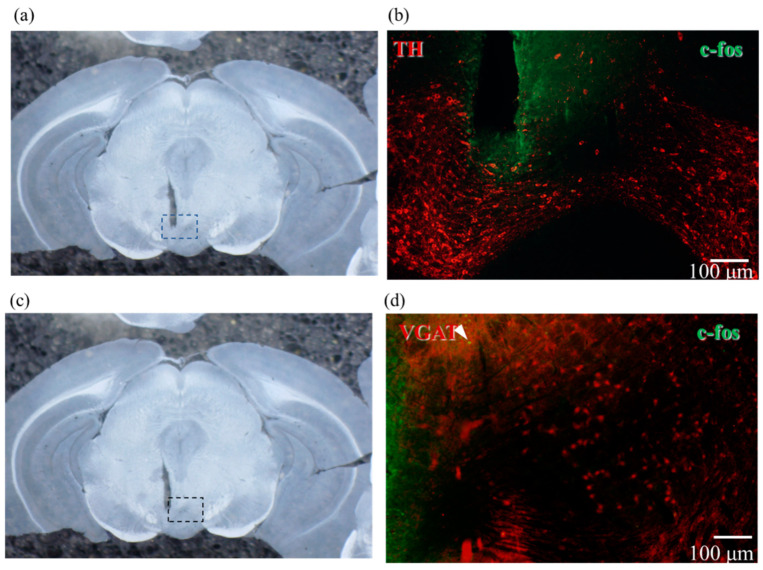
Fluorescence micrographs of double immunostaining of mouse brains not expressing ChrimsonR and photo-stimulated without expression of ChrimsonR and PS. The dotted box marks the area of fluorescence micrographs from the corresponding image on the left; the scale bar is 100 µm. (**a**,**c**) Photomicrograph of a coronal slice around 3.2 mm posterior of a wild-type mouse brain. (**b**) Fluorescence micrograph of the section shown in (**a**) with the dotted line enlarged. Red staining indicates dopaminergic neurons stained with anti-TH antibody, and green staining indicates active neurons stained with anti-c-fos antibody. Few neurons were stained with anti-c-fos antibody, indicating that dopaminergic neurons were not activated. (**d**) Fluorescence micrograph of the section shown in (**c**) with the dotted line enlarged. Red staining indicates GABAergic neurons stained with anti-VGAT antibody, and green staining indicates active neurons stained with anti-c-fos antibody. The double-stained area is only the arrowhead part, indicating that the GABAergic neuron is also less activated. Abbreviations: PS, photo-stimulation.

Figure 6a,c show an image of the coronal slice near the VTA in WT mice after PS. Figure 6b shows fluorescence microscopy images of immunostaining with TH antibody in red and c-fos antibody in green, unlike the other staining results in Figure 5, because ChrimsonR is not expressed here. There are few yellowish areas, indicating that there is almost no DA neuron activity in the VTA region, even after PS. 

Figure 6d shows fluorescence microscopy images of immunostaining with VGAT antibody in red and c-fos antibody in green. The only yellow area is indicated by an arrowhead, indicating that there is almost no GABAergic activity in the VTA region, even after PS.

## 3. Discussion

The dopaminergic network is part of the mesolimbic system and plays a significant role in the reward system. Although this network has been discussed in many papers, it has not yet been fully elucidated due to the complex interplay of other neural networks. In this study, we succeeded in selectively expressing ChrimsonR in DA neurons and used optogenetic technology to isolate and examine the relationship between DA and GABAergic neurons in the VTA. While it was difficult in older studies to stimulate neurons specifically using electrodes, using optogenetics we specifically stimulated DA neurons (and not GABA neurons) to clarify the role of GABA. We also examined the temporal changes of DA concentration in the NAcShell using microdialysis in addition to PS. Although microdialysis has poor temporal resolution, we believe that using DA receptor-specific fluorescence imaging such as GRAB-DA can increase confidence in the results. Future studies using PS will examine the control of physiological responses to alcohol or nicotine to elucidate the mechanisms of addiction.

In addition, DA release was impacted by PS frequency; the DA release was higher with 20 Hz pulse stimulation than with 2 Hz pulse stimulation. The power of the PS was the same as that of the total stimulation, i.e., 3 mA LED current and 30 s total stimulation time in both cases. It is not yet clear in detail whether these different pulse stimulation frequencies act on the DA neurons or on the GABAergic neurons that regulate the DA neurons. In this study, we used two frequencies, but we will use other frequencies as well in the future. In addition, as mentioned earlier, we will investigate the effect of PS specifically on GABAergic neurons. 

Our micro-LED array PS devices used in this study offer unique advantages over optical fibers and waveguide-type devices. Optical fibers can irradiate a range of 100–200 µm. However, our PS devices optically irradiate a range of 1.5–2.0 mm and stimulate large areas in the brain [18]. Furthermore, the implants in LED-based PS devices can be independently powered and are wireless, thus, they are completely unconstrained compared to those of optical fibers and waveguide devices.

We plan to expand our studies to further elucidate the role of the reward system in addictions, such as alcohol and nicotine.

## 4. Materials and Methods

### 4.1. Ethics Statement and Animal Protocol

The animal protocol was reviewed and approved by the Institutional Animal Care and Use Committee (IACUC) of the Nara Institute of Science and Technology (NAIST), (approval number: 2002; approval date 26 March 2020). Animals were housed in a facility that simulated a 12-h dark/light cycle environment. Food and water were provided freely.

In order to express ChrimsonR specifically in the DA neurons, we used the strain B6.SJL-Slc6a3tm1.1(cre)Bkmn/J mice (Jackson Lab, Bar Harbor, ME, USA), also known as DAT-Cre. The virus was EF1a-DIO-ChrimsonR-mRuby2-KV2.1 (Addgene, Watertown, MA, USA) injected into the VTA (AP: −3.5 mm, ML: 0.75 mm (left), DV: 4.5 mm) at an injection rate of 0.1 µL/min for 5 min. Then, to express ChrimsonR non-specifically in the DA neurons, we injected a CamKIIa-ChrimsonR-mScarlet-KV2.1 (Addgene) into the VTA of WT C57BL/6JJms Slc mice (Japan SLC, Inc., Hamamatsu, Shizuoka, Japan). Injection conditions were the same as for DAT-Cre mice. For the control group, C57BL/6JJms Slc mice not expressing ChrimsonR were used.

### 4.2. Photo-Stimulation (PS) Device and Fabrication

We have previously fabricated PS devices for rats and monkeys [17,18,22,23]. However, the device presented here for mice has been modified. The device is 300 µm wide, the same width as the LED, and increased the strength of the flexible printed circuits (FPC) in the harness. FPCs are made of polyimide, but are stiffened from the tip of the device to the harness to prevent them from bending. This enabled us to insert the device straight into the brain and capture the target VTA, which was about 4 mm from the brain surface. Additionally, the harness is straight to improve accuracy during implantation. The overall weight of the device was 0.01 g. The central emission wavelength of the LED (ES-AEHRAX12, Epistar Corporation, Taiwan) was set to 620 nm, the wavelength at which ChrimsonR is activated. In a typical biomedical implant, heat generation during PS to the brain tissue should be considered. However, in our previous study [14,18], we observed the heat generation from the PS and subsequent temperature rise from the LED was tolerable. In this study, we used the same settings and applied a current of 3 mA to the LEDs in this PS device, which activated ChrimsonR.

**Figure 7:**
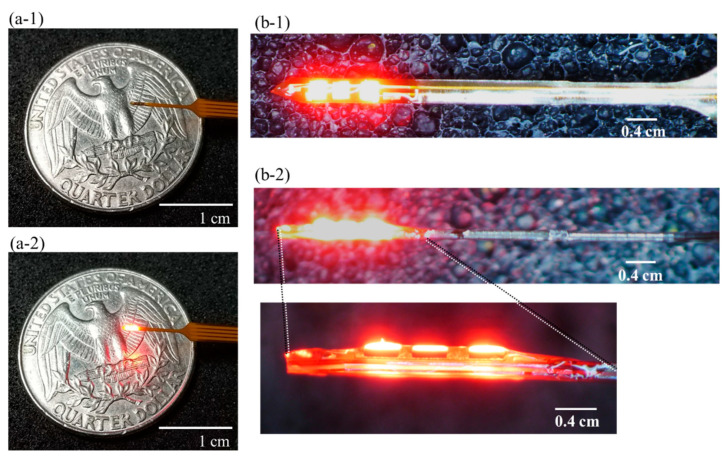
PS device. (**a-1**) Photo of the PS device with the LED not turned on. The weight of the device is 0.010 g. (**a-2**) Photo of the PS device when the LED is turned on. (**b-1**) Photo of the front side of the PS device when the LED is turned on. The LED (Epistar_ES-AEHRAX12) has a central wavelength of 620 nm, and its size is 300 × 300 (µm). The width of the device is 300 µm. (**b-2**) Photo of the side view of the device when the LED is turned on. The thickness of the device is 220 µm, including the LED and FPC. The lower photo in (**b-2**) is an enlarged photo of the area indicated by the line in the upper photo. Scale bars are 1 cm for (**a-1**,**a-2**), and 0.4 cm for (**b-1**,**b-2**). Abbreviations: FPC: flexible printed circuit, PS, photo-stimulation.

Three LEDs were bonded to the substrate with epoxy (Z-1, NISSIN RESIN Co., Ltd., Japan) and after further wire bonding, covered with additional epoxy to avoid damaging the tissue. Finally, biocompatible parylene-C was deposited for waterproofing and additional electrical insulation. The thickness of the deposited parylene film was about 5 µm. Figure 7(a-1,a-2) show the photos with the LED off and on, respectively. Figure 7(b-1) shows the front of the device, and Figure 7(b-2) shows the side of the device and its corresponding magnified photo. The cross-section including the LED and the harness is rigid and straight.

### 4.3. Stereotaxic Surgery

The surgical procedure of mice for AAV injection, PS device implantation, and microdialysis probe implantation was generally the same as in our previous paper [19,20]. Briefly, mice were anesthetized by IP injection with a triple anesthetic (medetomidine hydrochloride/mitazolam/betorphan, 0.75 mg/kg, 4.0 mg/kg, and 5.0 mg/kg, respectively) [24], placed on a stereotaxic instrument for mice (Narishige, Tokyo, Japan), and the scalp opened to expose the skull. The dura was incised with a pin and a PS device or microdialysis guide-tube is slowly inserted into position. The dura was covered with sealant (Kwik-Cast Sealant, World precision instruments), and the skull and devices are fixed with dental composite resin (Super-Bond Sun Medical, Moriyama, Shiga, Japan) to complete the procedure. Mice were awakened with an antagonist (medetomidine antisedan, 0.75 mg/kg).

The PS device was implanted in the mouse VTA (AP: −3.2 mm, ML: 0.6 mm (left), DV: 4.6 mm). The device was implanted in a median orientation so that the LED light illuminated the median side. During implantation, the axons of the DA neuron were extending in the rostral direction, and care was taken not to sever these axons.

The microdialysis guide-tube was implanted in the NAcShell (AP: 1.4 mm, ML: 0.5 mm (left), DV: 3.3 mm). A dummy probe was attached, and then the next day, replaced with a dialysis tube (EICOM, Kyoto, Japan) with a 1-mm-long dialysis membrane during the freely moving experiment.

### 4.4. PS and Microdialysis

Mice were allowed to recover for 24 h after implanting the PS device and microdialysis guide-tube, before performing PS and microdialysis experiments under freely moving conditions. Under isoflurane anesthesia, the connector of the PS device was connected to the cable, and the microdialysis dummy probe was exchanged with the dialysis probe and connected to the slipring and coiled tubing to allow free movement.

Subsequently, room temperature Ringer’s solution was allowed to perfuse through the NAcShell target site by coupling the probe inlet with a syringe pump (flow rate: 1 µL/min). Dialysates were collected every 15 min; an aliquot of 10 µL from the dialysates was injected into a high-performance liquid chromatographic column–electrochemical detector tandem (HPLC-ECD; EICOM, Kyoto, Japan) to measure the DA concentration. A calibration curve of DA concentration was constructed from the 1, 0.5, and 0.1 pg/µL DA standards. Base DA concentrations were designated via pre-stimulation measurement. Each PS session was completed within the initial 5 min of the 15-min dialysate collection periods. The frequency, duty cycle, and main interval of the pulse train were set before each session.

### 4.5. Experiments of PS and Bicuculline Injection

Microdialysis was performed in a free-moving mouse, and the DA concentration of the samples was measured by HPLC. We waited with measurement and observation until the DA concentration became constant and until the mice stabilized their behavior after anesthesia, about 2 h. Once these conditions were met, PS was performed. Bicuculline was injected into the peritoneal cavity 15 min before PS, and we adopted the result from reference [25] that bicuculline is effective as a GABA-_A_ receptor antagonist.

Samples were collected 6–8 times after PS. Since DA concentrations are expected to change from normal mouse behavior in addition to PS, the results of samples taken immediately after PS are mainly important.

Thereafter, mice were transcardially perfused with saline and then with 4% paraformaldehyde to fix the brain for subsequent histological and immunohistochemical analyses.

### 4.6. Immunostaining

To investigate the co-localization of ChrimsonR expressed in the VTA of DAT-Cre mice or WT mice with DA and GABAergic neurons, anti-TH antibody (anti-Tyrosine Hydroxylase antibody (Host: Rabbit, Polyclonal Antibody, AB152, Chemicon^®^, Merck Millipore, Burlington, MA, USA)) and anti-VGAT Antibody (Anti-Vesicular GABA Transporter Antibody (Host: Rabbit, Polyclonal), AB5062P, Sigma-Aldrich, Burlington, MA, USA)) were used for immunostaining. After completing the PS-microdialysis experiments, mice were anesthetized with urethane, perfusion-fixed, and the brains removed. Then, after immersion fixation in 4% PFA for 4–6 h, the brains were washed with PBS and prepared into 40 µm-thick coronal slices. The slice containing the photo-stimulated area was placed on a glass slide, and the surrounding PBS was absorbed with a filter paper, taking care not to break the section. Then, 50 µL of blocking solution (4% FBS/0.1% Triton X-100/0.05% NaN3/PBS) was poured onto the slice, placed in a moisture chamber, and incubated overnight at 4 °C. After this, the blocking solution was gently removed with filter paper and each slice was introduced to the primary antibody (either anti-TH antibody or anti-VGAT antibody, diluted in blocking solution at 1:1000 and 1:50, respectively). The 1 h incubation at room temperature was followed by five washes with PBS, each for 5 min.

Both anti-TH and anti-VGAT antibodies were Alexa Fluor^®^ 488 Goat IgG (H + L), Invitrogen) diluted in blocking solution at a concentration of 1:800. Finally, the slices were sealed with VECTASHIELD Mounting Medium (H-1000, Vector Laboratories), covered with a cover glass, and surrounded with sealant (Cover GripTM Coverslip Sealant, Cosmo Bio Co., Carlsbad, CA, USA). Each slice was photographed with a fluorescence microscope (Leica DMI6000B, Tokyo, Japan) and overlayed with ImageJ (an open-source, public domain image processing software: https://imagej.nih.gov/ij/, accessed 15 December 2021).

To examine neuronal activity, immunostaining was performed using anti-c-fos antibody (Host: Rabbit, Polyclonal Antibody, AB190289, abcam) as the primary antibody in the same manner as described above, and diluted to 1:500. Immunostaining was also performed on WT mice in the same way.

The primary antibody combination was Anti-TH antibody (Host: Rabbit, Polyclonal Antibody, AB152, 1:1000, Chemicon^®^) and Anti-c-fos antibody (Host: mouse, Monoclonal Antibody), ab208942, 1:500, abcam), Anti-VGAT antibody (Host: Rabbit, Polyclonal Antibody, AB5062P, Sigma-Aldrich, 1:50, Sigma-Aldrich) and Anti-c-fos antibody (Host: mouse, Monoclonal Antibody), ab208942, 1:500, abcam).

Secondary antibodies for both combinations were Alexa Fluor^®^ 568 Goat Anti-rabbit IgG (H+L) (Invitrogen, Waltham, MA, USA) and Alexa Fluor^®^ 488 Goat Anti-mouse IgG (H+L) (Invitrogen, Waltham, MA, USA), both diluted at 1:800.

### 4.7. Statistical Analysis

Statistical Analysis was performed using paired t-tests to identify statistically significant difference in DA concentrations between pre- and post-PS. The time-lapse graph of DA concentration every 15 min in Figure 4 shows the mean and standard deviation. Table 1 shows these together. A *p*-value less than 0.05 was considered significant.

## 5. Conclusions

In this study, we used a novel platform consisting of our previously developed micro-LED array PS device and a microdialysis probe to estimate DA release in the VTA of both ChrimsonR-expressing (non-specific) and DAT-Cre (DA-specific) mice. We measured DA release in the NAcShell in response to PS of VTA neurons and examined histochemically the effect of GABAergic neurons on DA neurons. The amount of DA released by NAcShell increased in response to PS of the VTA when the DA-specific ChrimsonR was expressed, whereas the amount of DA released by PS was almost unchanged when ChrimsonR was expressed non-specifically. However, when ChrimsonR was non-specifically expressed, microinjection of bicuculline into the VTA also significantly increased the amount of DA released at the NAcShell in response to PS of the VTA. The results of immunochemical staining confirm that GABAergic neurons in the VTA suppress DA activation, and also indicate that alterations in GABAergic neurons may have serious downstream effects on DA activity, NAcShell release, and neural adaptation of the VTA. Thus, it is significant that we investigated the relationship between mesolimbic DA neurons and GABAergic neurons in a neural-specific manner using optogenetics technology.

## Figures and Tables

**Table 1:** Mean and standard deviations of the DA release for the different experimental conditions shown in Figure 3.

Time (min)	DAT-ChrimsonR-2 Hz		DAT-ChrimsonR-20 Hz		WT-ChrimsonR-2 Hz		WT-ChrimsonR-20 Hz		WT-ChrimsonR-Bicuculline-2 Hz		WT-ChrimsonR-Bicuculline-20 Hz		Control-2 Hz		Control-20 Hz	
	N = 7		N = 6		N = 3		N = 3		N = 4		N = 3		N = 8		N = 6	
	Ave	STD	Ave	STD	Ave	STD	Ave	STD	Ave	STD	Ave	STD	Ave	STD	Ave	STD
0	1	0	1	0	1	0	1	0	1	0	1	0	1	0	1	0
15	1.187	0.096	1.506	0.405	1.120	0.112	0.967	0.075	1.276	0.146	1.47	0.083	1.040	0.080	1.148	0.301
30	0.994	0.145	1.208	0.293	1.023	0.201	1.021	0.175	1.114	0.105	1.400	0.495	0.976	0.176	1.166	0.321
45	0.987	0.132	1.091	0.205	0.899	0.129	0.823	0.017	0.958	0.166	1.172	0.447	0.905	0.160	1.133	0.331
60	0.970	0.189	0.944	0.170	0.926	0.226	0.795	0.142	0.878	0.115	1.364	0.475	0.936	0.128	1.065	0.315
75	0.963	0.204	0.978	0.199	1.010	0.169	0.702	0.006	0.754	0.180	1.171	0.407	0.978	0.149	1.092	0.291

## Data Availability

Data is available upon request.
